# *Streptococcus pneumoniae* endophthalmitis: clinical settings, antibiotic susceptibility, and visual outcomes

**DOI:** 10.1038/s41598-021-85456-3

**Published:** 2021-03-18

**Authors:** Kuan-Jen Chen, Ying-Jiun Chong, Ming-Hui Sun, Hung-Chi Chen, Laura Liu, Yen-Po Chen, Wei-Chi Wu, Eugene Yu-Chuan Kang, Chi-Chun Lai

**Affiliations:** 1 Department of Ophthalmology, Chang Gung Memorial Hospital, Taoyuan, Taiwan; 2 College of Medicine, Chang Gung University, Taoyuan, Taiwan; 3 Department of Ophthalmology, Penang General Hospital, George Town, Penang, Malaysia; 4 Department of Ophthalmology, Tucheng Municipal Hospital, New Taipei, Taiwan; 5 Department of Ophthalmology, Chang Gung Memorial Hospital, Keelung, Taiwan

**Keywords:** Outcomes research, Epidemiology

## Abstract

*Streptococcus pneumoniae* endophthalmitis is clinically more severe, more difficult to treat, and carry a higher risk of vision loss, evisceration, or enucleation. This study is to investigate the clinical settings, antibiotic susceptibility, and visual outcomes of *S. pneumoniae* endophthalmitis at a tertiary referral center in Taiwan*. S. pneumoniae* endophthalmitis was diagnosed in 38 eyes of 38 patients. The main clinical features were postcataract endophthalmitis (n = 13, 34%) and endophthalmitis associated with corneal ulcer (n = 12, 32%), trauma (n = 6, 16%), endogenous etiology (n = 4, 11%), trabeculectomy (n = 2, 5%), and pterygium excision-related scleral ulcer (n = 1, 3%). Presenting visual acuity ranged from counting fingers to no light perception. Pars plana vitrectomy with intravitreal antibiotics was performed in 17 eyes (39%) in primary or secondary treatments. *S. pneumoniae* isolates were susceptible to vancomycin (38/38, 100%), penicillin (37/38, 97%), ceftriaxone (37/38, 97%), cefuroxime (12/15, 80%), levofloxacin (13/15 ,87%), and moxifloxacin (15/17, 88%). Final visual acuity was better than 20/400 in 3 of 38 eyes (8%), 5/200 to hand motions in 3 eyes (8%), and light perception to no light perception in 32 eyes (84%). Ten eyes (26%) underwent evisceration or enucleation. Although *S. pneumoniae* isolates were susceptible to vancomycin, *S. pneumoniae* endophthalmitis had a very poor visual prognosis.

*Streptococcus pneumoniae* (pneumococcus) is a Gram-positive, spherical bacteria. They are usually found in pairs and are nonsporing and nonmotile. *S. pneumoniae* resides asymptomatically in healthy carriers, and they typically colonize the respiratory tract, sinuses, and nasal cavity. However, in individuals with compromised immune systems, such as elderly adults and young children, the bacterium may become pathogenic and spread to other locations to cause disease. *S. pneumoniae* has various virulence factors, including polysaccharide capsules, pneumolysin, neuraminidases, and zinc metalloproteinases. All these factors contribute to the severity of ocular infections^1,2^.

*S. pneumoniae* is still a main cause of ocular infectious diseases, such as conjunctivitis, keratitis, and endophthalmitis^1,2^. Although the endophthalmitis incidence is lower than that of conjunctivitis and keratitis, endophthalmitis is clinically more severe and more difficult to treat.

*S. pneumoniae* infection is a key cause of exogenous and endogenous endophthalmitis, and such infections have the worst visual sequelae among all organisms of endophthalmitis^3^. Of the 420 patients enrolled in the Endophthalmitis Vitrectomy Study, 291 had a positive culture, with a total of 323 growth isolates confirmed^4^. Of five patients, only two patients had visual acuity (VA) of 5/200 or better, and no patient had vision of 20/100 or better^3^.

In other studies of streptococcal endophthalmitis, *S. pneumoniae* led to the worst visual outcomes out of the culture-proved organisms examined^3,5–8^.

These outcomes include a lack of light perception and loss of eye globe despite antibiotic treatment and pars plana vitrectomy^5,6,9,10^. In other studies, the most common causes of *S. pneumoniae* endophthalmitis were surgery and trauma^5,9–12^. Furthermore, most cultured S. pneumoniae isolates cultured were susceptible to vancomycin^3,5–7,9,12–14^. This study investigated the clinical details, antibiotic susceptibility, and visual outcomes of patients with culture-proven endophthalmitis caused by *S. pneumoniae* infection at a tertiary referral center in Taiwan.

## Results

Thirty-eight eyes of 38 patients with culture-proven endophthalmitis caused by *S. pneumoniae* infection were identified. Tables 1 and 2 illustrate the demographics, systemic illnesses, clinical features, management details, and visual outcomes of patients with *S. pneumoniae* endophthalmitis. In terms of sex, 25 of the patients (66%) were male and 13 patients (34%) were female. The median age was 61.3 ± 26.7 years (range, 2 to 92 years). The systemic illnesses of interest included diabetes mellitus (in nine patients) and primary hypertension (in nine patients). The median follow-up interval was 21 months (range, 1 to 85 months).

**Table 1:** Demographics, clinical settings and features, managements, and outcomes of patients with *Streptococcus pneumoniae* endophthalmitis. *ACI* anterior chamber irrigation; *TAP*tap and injection.

Demographics	No. (%)	Clinical features	No. (%)
Patient number	38	Presenting visual acuity	
Eye affected	38	Counting fingers	4 (11%)
OD	22 (58%)	Hand motions	14 (37%)
OS	16 (42%)	Light perception	12 (32%)
Mean age, years	61.3 ± 26.7	No light perception	7 (18%)
**Gender**		Not available	1 (3%)
Male	25 (66%)	Hypopyon	34 (89%)
Female	13 (34%)	Fundus: invisible	38 (100%)
**Systemic illness**		**Management**	
Diabetes	9 (24%)	Primary	
Hypertension	9 (24%)	Vitrectomy	15 (39%)
Cancer	2 (5%)	Tap	15 (39%)
Coronary arterial disease	2 (5%)	Evisceration	6 (16%)
Tuberculosis	1 (3%)	ACI + TAP	2 (5%)
Aortic aneurysm	1 (3%)	Additional	
Ebstein's abnormality	1 (3%)	Tap	19 (50%)
Pneumonia	1 (3%)	Vitrectomy	5 (13%)
Infective endocarditis	1 (3%)	Evisceration/enucleation	4 (11%)
Unknown heart disease	1 (3%)	ACI + TAP	2 (5%)
**Clinical settings**		**Final visual acuity**	
Cataract	13 (34%)	≧ 10/20	1 (3%)
Corneal ulcer	12 (32%)	20/50–20/200	1 (3%)
Trauma	6 (16%)	19/200–20/400	1 (3%)
Endogenous	4 (11%)	4/200- Counting fingers	2 (5%)
Trabeculectomy	2 (5%)	Hand motions	1 (3%)
Pterygium excision-scleral ulcer	1 (3%)	Light perception	2 (5%)
		No light perception	30 (79%)

**Table 2:** Management and visual outcomes in *Streptococcus pneumoniae* endophthalmitis.

No	Sex	Age	Eye	Cause (interval between diagnosis and event, days)	Medical records	Initial VA	Primary treatment	Additional treatment	Final VA
1	F	84	OD	Cataract (3)	Gastric cancer	CF	PPV	TAP	NLP
2	F	75	OS	Cataract (1)	HTN	CF	TAP	TAP	NLP
3	F	83	OD	Cataract (2)	DM	HM	PPV	TAP	NLP
4	F	83	OD	Cataract (5)	DM, HTN, CAD	LP	PPV	PPV	CF
5	M	69	OS	Cataract (1)	HTN	LP	PPV	PPV	LP
6	M	84	OS	Cataract (3)	HTN, CAD	LP	PPV	TAP	NLP
7	F	60	OD	Cataract (2)	HTN	NLP	TAP	Evisceration	NLP
8	F	75	OD	Cataract (2)	DM, HTN	NLP	TAP	TAP	NLP
9	M	11	OD	Cataract (5)	RP, Ebstein's abnormality	LP	PPV	TAP	NLP
10	F	49	OS	Cataract (8)	HTN, heart disease	LP	TAP	Evisceration	NLP
11	M	75	OS	Cataract (3)		HM	PPV		20/50
12	M	79	OD	Cataract (3)	HTN, Aortic aneurysm	HM	PPV	TAP	20/400
13	M	71	OD	Cataract (2)	TB	HM	PPV	ACI + TAP	NLP
14	M	2	OD	PK- related CU ( 45)	Dermoid cyst	–	Evisceration		NLP
15	M	63	OS	PK- related CU ( 3 years)		HM	TAP	TAP	NLP
16	F	73	OS	PK- related CU ( 8 years)		HM	Evisceration		NLP
17	M	72	OD	PK- related CU (60)		LP	TAP	TAP	NLP
18	M	63	OS	PK- related CU (6 years)	DM	NLP	PPV	Evisceration	NLP
19	F	80	OD	PK- related CU (25)	DM, cancer	LP	PPV	TAP	NLP
20	M	63	OS	PK- related CU ( 5 years)		LP	Evisceration		NLP
21	F	41	OD	PK- related CU ( 5 years)		HM	TAP	TAP	NLP
22	F	69	OD	PK- related CU (7)	Lung cancer	LP	TAP	TAP	NLP
23	M	63	OS	Corneal ulcer		HM	TAP	TAP	NLP
24	M	80	OD	Corneal ulcer*^1^		NLP	Evisceration		NLP
25	M	69	OD	Corneal ulcer*^2^		HM	TAP	TAP	NLP
26	M	22	OS	Trauma/penetrating (3)		HM	PPV		HM
27	M	53	OD	Trauma/penetrating (2)	DM	LP	Evisceration		NLP
28	M	11	OD	Trauma/penetrating (4)		HM	PPV	TAP	CF
29	M	40	OD	Trauma/IOFB (1)		HM	PPV	TAP	NLP
30	M	35	OS	Trauma/IOFB (2)		HM	ACI + TAP	ACI + TAP	20/20
31	M	38	OS	Trauma/IOFB (1)		HM	TAP	PPV	NLP
32	M	78	OS	Endogenous	DM	LP	Evisceration		NLP
33	F	46	OS	Endogenous	DM	NLP	TAP	TAP	NLP
34	M	92	OD	Endogenous	DM, HTN, pneumonia	NLP	TAP	TAP	NLP
35	M	72	OD	Endogenous	IE	NLP	TAP	TAP	NLP
36	M	25	OD	Trabeculectomy (2 years)		CF	PPV	PPV	NLP
37	M	35	OD	Trabeculectomy (4 years)		LP	ACI + TAP	PPV	LP
38	F	74	OS	Scleral ulcer*^3^	Pterygium excision	CF	TAP	Enucleation	NLP

*ACI* anterior chamber irrigation; *CAD* coronary arterial disease; *CF* counting fingers; *CU* corneal ulcer; *DM* diabetes mellitus; *HM* hand motions; *HTN* hypertension; *IE* infective endocarditis; *IOFB* intraocular foreign body; *LP* light perception; *NLP* no light perception; *PK* penetrating keratoplasty; *PPV* pars plana vitrectomy; *TAP* tap and injection.

*Associated organisms: *Proteus mirabilis*^1^, *Serratia marcescens*^2^, and *Klebsiella oxytoca*^3^.

### Patient clinical settings and features

The main clinical features were postcataract endophthalmitis (n = 13, 34%) and endophthalmitis associated with corneal ulcer (n = 12, 32%), trauma (n = 6, 16%), endogenous etiology (n = 4, 11%), trabeculectomy (n = 2, 5%), and pterygium excision-related scleral ulcer (n = 1, 3%). Among 12 eyes with corneal ulcer–related endophthalmitis, nine (75%) of them had been subjected to penetrating keratoplasty (PK). Penetrating trauma caused endophthalmitis in three eyes, and an intraocular foreign body (IOFB) caused endophthalmitis in the remaining three eyes.

In the endogenous etiology group, endogenous causes included infective endocarditis and pneumonia, and two patients had unidentified primary sources of infection. One patient with scleral ulcer–related endophthalmitis underwent a complicated pterygium excision with subsequent scleral grafts. All patients with postcataract endophthalmitis developed acute-onset endophthalmitis (median, 3 days; range, 1 to 8 days). The two patients with trabeculectomy–associated etiologies developed endophthalmitis 2 years and 4 years posttrabeculectomy, respectively. The mean interval between trauma and diagnosis of endophthalmitis was 2 days (range, 1 to 4 days).

Severe blurring of vision and pain were symptoms observed for all 38 eyes (100%) and 35 of 38 eyes (92%), with mean durations of 2.2 and 2.0 days, respectively. Visual acuity (VA) test results were as follows: hand motions (HM; n = 14/38, 37%), light perception (LP; n = 12/38, 32%), no light perception (NLP; n = 7/38, 18%), counting fingers (4/38, 11%), and undetermined VA in one 2-year-old patient. Hypopyon was documented in 34 of 38 eyes (89%). Visualization in the posterior segment view was poor in all eyes due to severe anterior chamber inflammation and severe vitritis. The lacrimal sac findings did not indicate dacrocystitis in our series. Additionally, in the postcataract endophthalmitis subgroup, hypopyon was documented in 12 of 13 eyes (92%), and increased intraocular pressure (IOP > 21 mm Hg) was found in 8 of 13 eyes (62%). Presenting symptoms of pain and decreased VA were identified in 11 of 13 eyes (96%) and in 13 of 13 eyes (100%) and had mean durations of 1.9 and 2.1 days, respectively.

### Microbiology, antibiotic susceptibility testing, and minimal inhibitory concentration

*S. pneumoniae* infections were identified from vitreous samples from 35 (95%) of 37 patients and from anterior chamber taps in 19 patients (78%). Cultures from 35 eyes (92%) yielded a single microorganism. In three patients with polymicrobial endophthalmitis, *Proteus mirabilis*, *Klebsiella oxytoca*, and *Serratia marcescens* were identified*.* Antibiotic susceptibility testing and minimal inhibitory concentration (MIC) breakpoints of antibiotics are summarized in Tables 3 and 4.

**Table 3:** Antibiotic susceptibility testing of *Streptococcus pneumoniae* in patients with endophthalmitis.

Number (%)	Susceptible	Intermediate	Resistant
Vancomycin	38/38 (100%)		
Penicillin	37/38 (97%)		1/38 (3%)
Cefuroxime	12/15 (80%)		3/15 (20%)
Ceftriaxone	37/38 (97%)	1/38 (3%)	
Levofloxacin	13/15 (87%)		2/15 (13%)
Moxifloxacin	15/17 (88%)	2/17 (12%)

**Table 4:** Minimal inhibitory concentration of *Streptococcus pneumoniae* in patients with endophthalmitis. *MIC* minimal inhibitory concentration.

MIC (μg/ml)	0.125	0.25	0.5	1	2	4
Penicillin (n = 15)	1	4	5†	3	1*	1
Ceftriaxone (n = 16)	1	3	7†	4*	1	
Cefuroxime (n = 6)	1	1	2†		1	1*
Vancomycin (n = 6)		5†	1*			

*Indicates MIC_90_ values.

^†^Indicates MIC_50_ values.

### Management

Primary treatment consisted of a combination of vitreous tap and intravitreal antibiotics in 15 (39%) of 38 patients and pars plana vitrectomy (PPV) with intravitreal antibiotics in another 15 patients (39%). Follow-up treatments with vitreous tap and intravitreal antibiotics (TAP) were performed within 2 weeks of primary treatment in 19 (50%) of the 38 patients owing to clinically worsening inflammation and infection. One patient with traumatic endophthalmitis due to IOFB underwent removal of an anterior chamber IOFB and anterior chamber irrigation, and they were given antibiotics intravitreally. This patient was treated with a second course of anterior chamber irrigation with intravitreal antibiotics and eventually achieved 20/20 vision. Primary evisceration was performed on six eyes, and secondary evisceration or enucleation was performed on four eyes. In addition, intravitreal dexamethasone was administered to eight eyes (21%) during primary treatment.

### Final visual outcomes

Of the 38 eyes, final VA was 20/400 or better in three eyes (8%), 5/200 to HM in three eyes (8%), and LP to NLP in 32 eyes (84%). Of the three patients whose affected eyes achieved VA of 20/400 or better, one patient underwent removal of an anterior chamber IOFB (VA of 20/20), and patients had postcataract endophthalmitis (final VA, 20/50 and 20/400). Evisceration or enucleation was performed on 10 (26%) eyes.

### Statistical analysis

No significant difference in visual outcomes was observed between PPV and TAP groups (P = 0.99, Fischer’s exact test). Because of the relatively small number of eyes and the nonrandomized nature of this retrospective study, statistical conclusions concerning the differences between the clinical settings encountered and the treatment administered in this series cannot be drawn.

## Discussion

A comparison of previous and current studies is presented in Table 5 and includes clinical settings, the vancomycin sensitivity pattern, and final visual outcomes. The most common etiology in the current series was postoperative endophthalmitis; this finding is similar to those in studies conducted in the United States, Europe, Australia, and Thailand^5–10^. However, the most common cause in an Indian study was keratitis, followed by trauma and surgery^13^. In postoperative settings, the most common causes were cataract, trabeculectomy, and PK-related corneal ulcer. Because of the increasing trend toward the intravitreal administration of antivascular endothelial growth factor agents, streptococcus species such as viridans streptococci have become more common than *S. pneumoniae* as the causative organisms of intravitreal injection–related endophthalmitis^5^.

**Table 5:** Comparison in patients with Streptococcus pneumoniae in literature and present study.

Study no	Nationality	Year	No. of eyes	Etiology	No. of eyes	Vancomycin susceptibility no	Final VA ≧20/400 no. (%)	Evisceration/Enucleation no. (%)
1. Mao et al.^7^	US	1977–1990	6	NA		6/6 (100%)	2/6 (33%)	NA
2. Miller et al.^9^	US	1989–2003	27	All	27	27/27 (100%)	8/27 (30%)	2/27 (7%)
				Ocular surgery^1^	14		3 (21%)	
				Corneal stitch abscess	5		3 (60%)	
				Corneal ulcer	3		0	
				Trauma	3		2 (67%)	
				Endogenous	2		0	
3. EVS^3^	US	1990–1994	5	Cataract	5	5/5 (100%)	2/5 (40%)^2^	NA
4. Soriano et al.^10^	Spain	1986–2004	36	All	36	Not tested	NA	17/36 (47%)
				Cataract	18			
				Trauma	5			
				Glaucoma surgery	4			
				Radiotherapy	3			
				PK	3			
				Endogenous	2			
				Corneal abscess	1			
5. Kurniawan et al.^6^	Australia	1997–2012	23	NA		23/23 (100%)	4/20(20%)^3^	9/20(45%)
6. Kuriyan et al.^5^	US	2000–2011	13	All	13	13/13(100%)	5/13 (38%)	NA
				Cataract	4			
				Bleb	4			
				GDD	2			
				PK	1			
				IVI	1			
				Corneal ulcer	1			
7. Dave et al.^11^	India	2004–2017	68	NA	68	51/52 (98%)	0	68/68 (100%)
8. Jindal et al.^13^	India	2006–2013	105	Trauma	105	> 98%	NA	NA
9. Dave et al.^12^	India	2006–2018	36	Endogenous	36	> 98%	NA	NA
10. Yospaiboon et al.^8^	Thailand	2012–2016	20	All	20	NA	NA	NA
				Postoperative	11			
				Trauma	4			
				Keratitis	2			
				Undetermined	3			
11. Chen et al. (current study)	Taiwan	2000–2019	38	All	38	38/38 (100%)	3/37 (8%)	10/38 (26%)
				Cataract	13		2/13 (15%)	
				PK- related ulcer	9		0	
				Corneal ulcer	3		0	
				Trauma	6		1/6 (17%)	
				Endogenous	4		0	
				Trabeculectomy	2		0	
				Scleral ulcer	1		0	

*GDD* glaucoma drainage device; *IVI* intravitreal injection; *NA* not available; *PK* penetrating keratoplasty.

^1^Ocular surgery: Cataract extraction (5), bleb-associated (4), lensectomy/anterior vitrectomy (1), bleb needling (1), suture removal (1), penetrating keratoplasty (1), glaucoma drainage device (1).

^2^Final VA ≧5/200.

^3^Final VA > 20/200.

*S. pneumoniae* is generally sensitive to vancomycin, penicillin, ceftriaxone, and fluoroquinolone. In Western countries, most related case series reported that it had susceptibility to vancomycin (100%)^4,5,7,9,10^. However, in India, some *S. pneumoniae* isolates showed resistance to vancomycin^12–14^. In this study and studies from Australia and Thailand, all *S. pneumoniae* isolates were sensitive to vancomycin (100%)^6,8^. Although fluoroquinolones, such as levofloxacin and moxifloxacin, are widely used in the treatment or prophylaxis of endophthalmitis, some *S. pneumoniae* isolates have been reported to still be resistant to these treatments^9,12^. In our study, some *S. pneumoniae* isolates were partially resistant to moxifloxacin or levofloxacin. Our study demonstrated that the MIC_90_ of vancomycin for *S. pneumoniae* was 0.5 μg/mL. We propose that vancomycin be continued as the first-line intravitreal antibiotic in the treatment of *S. pneumoniae* endophthalmitis.

Studies on endophthalmitis caused by *S. pneumoniae* infection showed a higher rate of poor visual and anatomical outcomes in those with the disease. US case series^3,5,7,9^ have found better visual outcomes than in Asian, European, and Austrian studies^6,8,10,12–14^. In the present study, only three eyes (3 out of 38, 8%) achieved vision of 20/400 or better. Patients with endophthalmitis caused by *S. pneumoniae* or *P. aeruginosa* infection have a higher risk of requiring evisceration or enucleation^13,15^. Miller et al., in their study on *S. pneumoniae* endophthalmitis, reported that 3 out of 27 (11%) eyes required evisceration^9^. In Spanish and Indian studies, higher rates of evisceration of eyes affected by *S. pneumoniae* endophthalmitis were identified^10,13^. Soriano et al.^10^ reported an evisceration rate of 47% in 36 cases of *S. pneumoniae* endophthalmitis. However, our study demonstrated an evisceration or enucleation rate of 26% in 38 cases of *S. pneumoniae* endophthalmitis.

Early detection and treatment are the best approaches to improving final visual outcomes in patients with endophthalmitis. Although some case series have shown no statistical difference between vitrectomy and TAP, early PPV still offers a higher likelihood of achieving a more favorable visual prognosis^6,9,10^. The need for a higher number of intravitreal injections was significantly associated with a poor final VA^7^. The rate of evisceration or enucleation in patients with endophthalmitis caused by *S. pneumoniae* did not decrease in recent studies^5,6,8,13,14^. Chen et al.^16^ demonstrated that prevention of evisceration or enucleation in patients with endogenous bacterial panophthalmitis, no light perception, and scleral abscess can be achieved through multiple intravitreal and periocular injections of antibiotics and dexamethasone. No eye required evisceration or enucleation. Multiple intravitreal and periocular injections of antibiotics and dexamethasone could be an alternative to evisceration or enucleation in such cases.

This study has several limitations. First, the study was retrospective. Second, a few subgroups, such as those with traumatic, trabeculectomy-related, and endogenous *S. pneumoniae* endophthalmitis, had a relatively small number of patients. This made statistical analysis difficult to perform. Third, only a few antibiotics and samples were subjected to antibiotic susceptibility testing. However, vancomycin is regularly tested by the laboratory department of our hospital. Because vancomycin is a routine antimicrobial agent used in treating Gram-positive bacterial endophthalmitis, our results still offer useful information. Despite these limitations, this study provided data on antibiotic susceptibility patterns and visual outcomes of endophthalmitis caused by *S. pneumoniae*.

In conclusion, the most common cause in *S. pneumoniae* endophthalmitis infection was postcataract status. Although all *S. pneumoniae* isolates were susceptible to vancomycin, patients with *S. pneumoniae* endophthalmitis typically have a very poor visual prognosis. Further studies are necessary to ensure better prevention and management of endophthalmitis caused by *S. pneumoniae*; this could include preventing he emergence of endophthalmitis and examining the effectiveness of prompt, aggressive medical and surgical interventions for the management of endophthalmitis.

## Methods

The Institutional Review Board of Chang Gung Memorial Hospital in Taoyuan, Taiwan approved this retrospective study protocol and waived the need for written informed consent from the study patients. All clinical procedures were conducted according to the principles of the Declaration of Helsinki. The medical records of all patients treated for endophthalmitis caused by *S. pneumoniae* infection were reviewed at a tertiary referral center between April 2019 and January 2000. The inclusion criteria were as follows: (1) Positive *S. pneumoniae* cultures from intraocular fluid (aqueous humor and vitreous) were identified in patients with exogenous endophthalmitis, including after surgery and trauma; (2) For patients with endogenous endophthalmitis, their bodily fluids were cultured with growths of *S. pneumoniae*. (3) The patients received follow-ups for at least 3 months unless, based on their final best-corrected VA, they had NLP. Data collected and reviewed included demographic information, medical history, presenting signs and symptoms, duration of symptoms before diagnosis of endophthalmitis, intervals between event and diagnosis of endophthalmitis, culture sites, antibiotic sensitivities and resistance patterns, management administered, and final VA.

Intraocular fluids were collected and plated on blood agar (5% sheep), chocolate agar, fungus culture media, and thioglycolate broth. All microbiologic testing was performed at the Microbiology Department of Chang Gung Memorial Hospital, Taoyuan, Taiwan. Bacterial culture isolates were identified through conventional microbiological methods (2000–2013) and matrix-assisted laser desorption ionization time of flight mass spectrometry (MALDI-TOF–MS; 2014–2019). Conventional microbiological methods included Gram staining and biochemical tests. Gram staining revealed Gram-positive, lancet-shaped cocci. Positive reactions included optochin, bile solubility, inulin and alfa hemolysis, and negative reactions included catalase and oxidase. When the isolates were identified through MALDI-TOF MS (Bruker Daltonics, Germany), the microbial film was overlaid with 1 μL of 70% formic acid. After the sample had dried, the film was overlaid with 1 μL of the matrix solution (50% acetonitrile containing 1% a-cyano-4-hydroxycinnamic acid and 2.5% trifluoroacetic acid). After the sample had dried again, microorganism identification and data analysis were performed, for which the Bruker LT Microflex MALDI-TOF MS and Bruker Biotyper 3.0 system software programs were used. All analyses were performed using standard methods. An identification was considered successful at the species level when the score exceeded 2.0. The isolates were tested for susceptibility to various antibiotics using the Kirby–Bauer Disc diffusion method on Mueller Hinton blood agar. The MIC breakpoints were determined based on the 0.5 McFarland standards by using a microdilution broth and the E-test method. The Clinical and Laboratory Standards Institute (Wayne, PA) standards were used for interpretation and quality control for each corresponding year^17^. Because of the limitations inherent in a retrospective study, the antibiotic susceptibility testing of *S. pneumoniae* isolates was performed in some selected antibiotics, including vancomycin, penicillin, ceftriaxone, levofloxacin, and moxifloxacin. Some *S. pneumoniae* isolates were tested to determine the relevant MIC of antibiotics such as penicillin, ceftriaxone, cefuroxime, and vancomycin.

The treatment strategies were determined by the individual treating physicians and did not follow a standardized protocol. Before *S. pneumoniae* was cultured, intravitreal antibiotics included vancomycin with either ceftazidime or amikacin. After positive cultures of *S. pneumoniae* isolates and antibiotic susceptibility testing results were obtained, intravitreal vancomycin was administered. The doses of intravitreal agents were as follows: vancomycin, 1 mg/0.1 mL; amikacin, 0.25–0.4 mg/0.1 mL; ceftazidime, 2.25 mg/0.1 mL; and dexamethasone, 0.4 mg/0.1 mL. Tap with intravitreal antibiotics was treated as follows: (1) Corneal clarity was not suitable for PPV. (2) Patients with poor systemic condition were not suitable for PPV. (3) Patients refused PPV because of poor visual prognosis . Pars plana vitrectomy was performed as follows: (1) corneal clarity possible for PPV, and (2) poor response to initial tap treatment. Enucleation or evisceration was performed when the globe was not salvageable due to corneal or scleral necrotic laceration. Poor visual outcomes were defined by VA worse than 20/400, whereas favorable prognoses were defined by VA of 20/400 or better. Statistical analysis was performed using SPSS for Windows version 23 (SPSS Science, Chicago, IL, USA).
